# Difficulties experienced by health care professionals who performed home visits to screen for postpartum depression during the COVID-19 pandemic: a qualitative study in Japan

**DOI:** 10.1186/s12913-023-09687-y

**Published:** 2023-06-22

**Authors:** Aiko Furudate, Kenzo Takahashi, Kentaro Kinjo

**Affiliations:** Teikyo University Graduate School of Public Health, 2-11-1 Kaga, Itabashi-ku, Tokyo, 173-8625 Japan

**Keywords:** Health care professionals, Postpartum depression, COVID-19, Maternal–child health service

## Abstract

**Background:**

Postpartum depression is a risk factor for suicide and maltreatment of children, and its early detection and appropriate intervention are issues to be resolved. In Japan, local governments are working to detect postpartum depression early by conducting home visits to families with infants within 4 months postpartum, but home-visit professionals have faced new difficulties due to the coronavirus disease 2019 (COVID-19) pandemic that started in 2020. The purpose of this study was to clarify the difficulties experienced by health care professionals who perform home visits to screen for postpartum depression.

**Methods:**

Focus-group interviews were conducted during the COVID-19 pandemic with health care professionals (n = 13) who make postpartum home visits to families with infants within 4 months. Data were analyzed using thematic analysis.

**Results:**

Four main categories were identified that describe the difficulties experienced by health care professionals: “Lack of support for partners,” “Difficulty in talking face-to-face,” “Inability to offer family assistance,” and “Anxiety about being a source of infection.”

**Conclusions:**

This study shed light on the difficulties faced by professionals in supporting mothers and children in the community during the COVID-19 pandemic. Although these difficulties were considered to have become apparent during the pandemic, the results may offer an important perspective for postpartum mental health support even after the pandemic ends. Accordingly, it may be necessary for these professionals to receive supported through multidisciplinary collaboration in order to improve postpartum care in the community.

## Background

The World Health Organization (WHO), in cooperation with several other international organizations, published a report that called for a reduction in the global maternal mortality rate to less than 70 per 100,000 live births by 2030 [1]. The maternal mortality rate in Japan was 3.3 per 100,000 live births in 2019, which is low compared with the rest of the world. However, the maternal suicide rate was extremely high at 8.7 per 100,000 live births in the 23 wards of Tokyo during the 10-year period from 2005 to 2014 [2].

Depressive disorder is one of the risk factors of prenatal and postpartum suicidal ideation, with a higher prevalence of depression in women who attempted suicide postpartum than during pregnancy [3]. Meanwhile, postpartum depression is a risk factor for suicide [4] and maltreatment of children [5], and thus early detection of postpartum mental health problems and appropriate intervention are essential for both mothers and child health services. In addition, bonding disorders associated with postpartum depression [6] and maltreatment of children (e.g., neglect) are also major problems. Therefore, countermeasures against postpartum depression are considered an urgent public health issue.

A previous study reported that the continuum of care for maternal, neonatal, and child health was associated with a reduction of 500,000 maternal deaths, 4,000,000 neonatal deaths, and 6,000,000 child deaths globally each year and suggested that expanding postnatal care might result in further reductions [7]. The WHO recommends a minimum of four postnatal care contacts (at least 24 h after birth, between 48 and 72 h, between 7 and 14 days, and during week 6 after birth) as well as home visits by health professionals [8].

In Japan, where approximately 98% of births take place in hospitals or clinics and mothers are often discharged about a week after delivery, each municipality conducts home visits to families with infants within 4 months of birth through a welfare program called Konnichiwa Akachan Jigyo [9]. In this program, public health nurses, midwives, or other health care professionals visit all families with infants in the area, interview them regarding their concerns about childrearing, and provide information on childrearing support provided by the municipality. At the same time, they screen for postpartum depression, using the Japanese version of the Edinburgh Postpartum Depression Scale (EPDS) [10, 11]. A study found that continuous support provided by integrated mental health care through a multidisciplinary maternal–child health service in the community improved the mental health of postpartum women and increased their access to more health services [12]. Their findings suggest the importance of maternal–child health services involving home visits.

The global coronavirus disease 2019 (COVID-19) pandemic has drastically changed people’s lives and affected the maternal and child health care field. In Japan, people were asked to change their lifestyle to prevent the spread of infection by avoiding the “Three Cs” (closed spaces, crowded places, close-contact settings [13]), practicing physical distancing, wearing masks, and washing their hands. A state of emergency was declared four times from April 2020 through June 2022 by the Japanese government, which urged citizens to reduce person-to-person contact, including refraining from unnecessary outings and travel between prefectures, and requested that some business facilities close temporarily or reduce their business hours. At childbirth facilities, childbirth preparation classes, postpartum visits by family, the attendance of partners at the birth, and the acceptance of pregnant women relocating from other prefectures were prohibited [14].

In Japan, it is customary for pregnant women to return to their parents’ home to prepare for childbirth. This is known as *sato-gaeri bunben* (hometown childbirth) [15] and is a traditional form of childbirth support in Japan [16]. More recently, an increasing number of local governments have begun to provide public subsidies to postpartum care facilities as an alternative public service to *sato-gaeri bunben*. However, there was a period when many mothers were unable to give birth in their hometown because of calls to refrain from travel between prefectures and because many postpartum care facilities stopped accepting mothers from other prefectures. As reported by a recent study in Japan, 30% of pregnant women who hoped to return to their hometown for childbirth could not do so because of the COVID-19 pandemic [17]. These women are likely to have experienced more stress than those who were able to return to their hometown for the delivery as scheduled. During the COVID-19 pandemic, postpartum mothers might have experienced an increase in anxiety due to the lack of social support in Japan. The prevalence of postpartum depression among mothers who gave birth and took care of children during the pandemic was 28.7% [18], double the prevalence of (14.3%) reported in a meta-analysis of Japanese women published in 2020 [19]. According to that report, this change may have been due to the fact that an increasing number of mothers were unable to go out as much as they would have liked, were unable to return to their parents’ home or ask their parents for help, and were unable to meet with friends or interact with others who gave birth around the same time.

Under such circumstances, health care professionals visiting the homes of families with infants are likely to experience new and unprecedented difficulties. In fact, it has been reported that at least 60% of municipal public health nurses who communicate directly with community residents had experienced mental health difficulties [20]. However, few studies have investigated the difficulties experienced by health care professionals who visited the homes of families with infants during the COVID-19 pandemic. A study conducted in China reported that health care workers employed in hospitals during the COVID-19 pandemic needed support to prevent exhaustion [21]. We believe that providing support that addresses the difficulties and challenges faced by health care professionals is essential to prevent turnover due to exhaustion, thereby enabling them to continue to provide support for postpartum mothers. To that end, we considered it necessary to conduct a preliminary study to identify the challenges faced by health professionals who visit postpartum mothers directly and to examine whether solving these challenges in the future would improve the care and mental health of postpartum mothers in the community.

Accordingly, this study conducted focus-group interviews with health care professionals who make home visits to families with infants with the aim of clarifying the difficulties they have faced during the COVID-19 pandemic and considering how to provide better support for mothers and infants in the future.

## Methods

### Study design

This is an exploratory qualitative study using focus-group interviews. The study is reported following the Standards for Reporting Qualitative Research framework [22].

### Participants

Participants were 13 of the 15 health care professionals (public health nurses, midwives, and nurses) employed by City A who conduct home visits to all families with infants. These 13 participants were selected because they were available to participate in interviews conducted at monthly meetings (Table 2). City A is located in the Kanto region of Japan and has a population of approximately 170,000 with about 900 births per year. City A has four maternity facilities and 17 obstetricians.

### Data collection

This qualitative study was conducted by means of focus group interviews. As a research question, we attempted to identify what difficulties health professionals who visited the homes of postpartum mothers with children up to 4 months of age in City A had experienced during the COVID-19 pandemic.

Participants were divided into two groups according to their work schedule. The first group (n = 6) attended an interview session on November 11, 2021 that lasted 81 min and 53 s, and the second group (n = 7) attended an interview session on December 16, 2021 that lasted 104 min and 49 s. A state of emergency had been declared four times since April 2020 in response to the COVID-19 pandemic, with the fourth state of emergency being lifted on September 30, 2021. During the group interviews, we used an interview guide that included the following questions: Do you have any difficulties at work? What has changed or been difficult for you in providing support for mothers since the start of the COVID-19 pandemic? Which cases did you find especially difficult? (Table 1)

**Table 1:** Interview Guide

1	Self-introduction
2	What kinds of mothers do you worry about most?
3	What do you think postpartum mothers have the most trouble with?
4	Do you have any difficulties at work?
5	What has changed or been difficult for you in providing support for mothers since the start of the COVID-19 pandemic?
6	Which cases did you find especially difficult?”

Interviews were conducted in a well-ventilated room in a municipal facility where privacy could be ensured; the interviews were recorded with a digital voice recorder after obtaining the permission of the study participants. Interviews for each group started with an introduction and then proceeded with questions for participants about their difficulties in providing support to mothers and their children during the pandemic.

### Data analysis

The interview recordings were transcribed verbatim. First, the first author (AF) read all of the transcripts. Then coding of the transcripts was performed to generate a preliminary list of codes. The transcripts were imported to the analytical software MAXQDA 2022 (VERBI GmbH, Berlin, Germany) and codes were generated to systematically capture interesting aspects of the data across the entire dataset. We used a thematic analysis approach because it allows for a flexible and nuanced account of the data [23]. Codes with similar content were grouped and labeled, and then subcategories (indicated with < >) and categories (indicated with “ ”) were generated. Analysis was performed with the input of multiple researchers in order to ensure validity. One author (AF) performed the preliminary analysis and another author (KK) served as a second, independent coder of the data. The initial coding was then reviewed and compared. The codes were further reviewed and refined through discussion until consensus was reached among all authors. To analyze the relationships between categories, one author (KK) created a preliminary diagram of the codes, subcategories, and categories. After all authors had reviewed and discussed all the codes and categories, a refined diagram was completed based on saturated logic.

### Ethical considerations

This study was approved by the Teikyo University Medical Research Ethics Committee (Teirin 21–132), and all methods were performed in accordance with the Declaration of Helsinki. Consent to participate was obtained in writing. Participants were reminded that they could stop their participation in the interview at any time without giving a reason. Identifying information was removed from the transcripts to protect participants’ anonymity.

## Results

### Participants

Of the 13 study participants, 9 were public health nurses, 3 were midwives, and 1 was a nurse. They had an average of 10.8 years (range 8 months to 30 years) of experience in maternal and child health activities as municipal employees. They ranged in age from their 20s to their 50s, and all were women. The participant characteristics are presented in Table 2.

**Table 2:** Characteristics of participants

Variables	N
Occupation	
Public health nurse	9
Midwife	3
Nurse	1
**Experience in maternal and child health activities**	
< 1 year	1
1–10 years	8
11–20 years	1
> 20 years	3
**Age**	
20s	2
30s	5
40s	2
50s	4
**Sex**	
Female	13
Male	0

### Difficulties experienced by healthcare professionals who visited the homes of families with infants to screen for postpartum depression during the COVID-19 pandemic

Focus groups interviews were performed. The total interview time was 186 min and 42 s, and the total word count of the verbatim transcript was 48,460 words. From these sentences, 175 codes were generated using the inductive coding approach, following the principles of reflexive thematic analysis [23]. Furthermore, the co-authors decided on 11 subcategories. After discussing the subcategories and codes based on saturated logic, a qualitative analysis of the interview data was performed, which led to the identification of four categories of difficulties experienced by healthcare professionals who visited the homes of families with infants during the COVID-19 pandemic: “Lack of support for partners,” “Difficulty in talking face-to-face,” “Inability to offer family assistance,” and “Anxiety about being a source of infection.” (Table 3).

**Table 3:** Overview of identified themes

Main category	Sub-categories
1. Lack of support for partners	1.1 Partners are stay home more often
	1.2 No tools to assess partners
	1.3 No opportunity to talk with partners
2. Difficulty in talking face to face	2.1 Refusal to allow home visits
	2.2 Mothers are overly sensitive
3. Inability to offer family assistance	3.1 Inability to ask for support from one’s parents
	3.2 Inability to use postpartum care facilities
	3.3 No place to leave the baby to give the mother time to rest
4. Anxiety about being a source of infection	4.1 Stress about being a potential source of infection
	4.2 Stress regarding close physical contact
	4.3 Concern about infection control during visits

## Category 1: lack of support for partners

After the start of the COVID-19 pandemic, healthcare professionals noticed that mothers and their partners were spending more time together at home. At the same time, providers considered it important to assess partners’ attitudes toward childcare and health status, but they found it difficult to talk with the partners without standard assessment tools. This category comprised three subcategories:

<Partners are staying home more often>.

Due to restrictions on going out during the COVID-19 pandemic as well as the increase in people working from home, partners were spending more time at home. However, they seemed to struggle with uncertainty about how they should assist mothers and children. Health care providers had difficulty regarding how to intervene.

I don’t have the necessary tools, and I’m still a little unsure of what her partner thinks and how he perceives the birth of his child.

<No tools to assess partners>.

Although health care providers were able to assess mothers and children during visits by using EPDS, physical measurements, and interviews, the lack of assessment tools for the partner present made it difficult for them to deal with the situation.

She seems to need her partner’s help to make it work, but he is already in a situation where he is struggling.

<No opportunity to talk with partners>.

At the time of the visit, the partner was at home but not present in the room, sometimes causing the health care worker to feel like their presence was an intrusion when they visited the mother and child. Health care providers also experienced difficulties in communicating with the partners.

There have been cases where a partner has left because of my presence, saying, “It’s okay, you’re just going to say bad things about me anyway,” and I alienated him by taking the mother’s side.

## Category 2: Difficulty in talking face to face

Against the background of physical distancing during the COVID-19 pandemic, mothers sometimes refused to allow home visits and health care professionals could not talk face-to-face so the meeting took place over the phone instead. This category comprised two subcategories:

<Refusal to allow home visits>.

In the early stages of the COVID-19 pandemic, the disease was considered highly contagious and potentially fatal, so visits were considered to increase the risk of infection. Therefore, an increasing number of mothers did not wish to receive home visits. Health care providers found it difficult and challenging to assess maternal and child health through telephone interviews.

Since COVID-19, I have been getting a lot of rejections when I call to make an appointment for a visit. At the beginning of the state of emergency, I didn’t visit their home and instead talked with mothers on the phone, but it was very difficult for me to judge the severity of the situation because I couldn’t see her face and couldn’t ask her questions from the EPDS in detail.

<Mothers are overly sensitive>.

The mother was extremely sensitive to the possibility of getting COVID-19 from the health care worker during the visit.

When I placed my work bag on the doorstep, they complained that it was dirty.

## Category 3: inability to offer family assistance

The health care professionals were concerned about mothers who were unable to return to their hometown to prepare for childbirth or could not ask their own mother for help because of the COVID-19 pandemic and the calls to refrain from travel between prefectures. Also, some postpartum care facilities had stopped accepting mothers at times, and the health care professionals felt that they could no longer encourage mothers to get help from family members or health care facilities at a time when the mothers should be getting some rest. This category comprised three subcategories:

<Inability to ask for support from parents>.

In Japan, mothers often give birth and spend the postpartum period at or near their parents’ home (i.e., hometown childbirth). When the COVID-19 pandemic made it difficult for mothers and children to travel and there were no family members nearby to take care of the children, the mothers were considered to be at higher risk of childcare stress.

It is difficult to find a place to leave the children for some time so that the mother can rest, unless you have a family member who can take care of them, and this is one of the unfortunate aspects of the COVID-19 pandemic.

<Inability to use postpartum care facilities>.

The COVID-19 pandemic prevented mothers and children from staying overnight because home care was encouraged and postpartum care facilities were closed.

Major facilities were closed for about 6 months in August, so there were people who wanted to stay overnight and rest during this time but were unable to do so.

<No place to leave the baby at night>.

The COVID-19 pandemic placed a greater burden on mothers to take care of their children at night by themselves when there were no family members nearby or the facility was closed. Healthcare providers experienced difficulties in providing support for them.

It is a great burden to have to take care of the children by myself at night, and they cannot sleep. It’s not easy unless you have a family member that you can leave your child with.

Even if we introduce them to the support services, we cannot take over childcare for them. It does not eliminate their burden.

## Category 4: anxiety about being a source of infection

Healthcare professionals were under increasing pressure not to be a source of infection during home visits. In particular, they felt anxious about holding the baby and providing breastfeeding care that involved direct contact. This category comprised three subcategories: <Stress about being a potential source of infection>.

During home visits, health care workers were stressed because they had to take care not to infect the mother and child.

Medical workers were more stressed about being a source of infection than being infected themselves, and I felt the same stress.

<Stress regarding close physical contact>.

Although the health of the mother and child is usually investigated through contact with the child at home, the health care workers felt stressed because they could not make contact so as to prevent the spread of COVID-19.

I couldn’t have direct contact with children, such as holding the infant or their siblings, during the home visit like I used to. Normally, I would say, ‘It’s OK, mom, I’ve got him,’ and I would hold him while recording or while having her fill out the Edinburgh questionnaire, but I couldn’t do that.

<Concern about infection control during visits>.

At the time of the visit, health care workers were concerned about the extent to which they should take infection control measures for COVID-19.

I was told that my bag was dirty when I put it in the doorway.

### Relationships among the categories

It was difficult to examine the relationships between categories through code relationship analysis using MAXQDA because the categories were too close together. Therefore, it was necessary to review and discuss the verbatim transcripts, codes, and subcategories. To analyze the relationships between categories, authors analyzed the data while considered similarities and dissimilarities, not conducting a simple descriptive analysis. Based on the reviews and discussions, the most important categories for health care providers visiting the home were considered to be category 2 (difficulty in talking face to face) and category 4 (anxiety about being a source of infection). In other words, the factors in categories 2 and 4 were considered to have made the visit more difficult and were thought to interact and influence the other two categories. With category 1 (Lack of support for partners), those three categories are thought to ultimately affect category 3 (Inability to offer family assistance).

## Discussion

This study revealed four categories of difficulties for healthcare professionals who visited families with infants to screen for postpartum depression during the COVID-19 pandemic: lack of support for partners, inability to meet and talk directly with mothers, inability to offer family assistance, and anxiety about being a source of infection. The relationships between the categories suggested that categories 2 and 4 in particular (Difficulty in talking face to face and Anxiety about being a source of infection), which were new concerns caused by the COVID-19 pandemic, were top of mind and were thought to affect the other categories. Along with the new finding that mothers felt unsupported by their partners, which had not been discussed during visits before, the ultimate finding was that they felt difficulties regarding the lack of support for their families.

In a Japanese study, the incidence of male depression at 1 and 6 months postpartum was 11.2% and 12%, respectively, which is about the same as that of maternal postpartum depression [24]. Although healthcare providers were aware that the incidence of male postpartum depression was increasing, they found it difficult to assess the mental health of the partners. Some studies have shown that the EPDS is a useful measure of postpartum depression in fathers [25], but there is still no consensus on the cutoff value. In the future, it will be necessary to establish a cutoff value for paternal EDPS in accordance with the situation in each country, and to use the same standard to evaluate the situation and take appropriate countermeasures. In a meta-analysis of five qualitative studies, Kido et al. reported that support for fathers who are aware of postpartum depression is inadequate, that male gender is a barrier to seeking help, and that fatherhood motivates fathers to take action to overcome postpartum depression [26]. Takehara et al. also pointed to the lack of easy contact with fathers after childbirth and the rapidly increasing role expected of fathers after childbirth in the social context as obstacles to assessing postpartum depression in fathers [27]. As expectations for paternal participation in childcare continue to increase, everyone must recognize that postpartum depression can occur in fathers as well as in mothers, and efforts must be made to provide early screenings and interventions, using appropriate assessment instruments such as the EPDS.

During home visits in the present study,, mothers talked at length about not only childcare but also things they would normally discuss with friends. Tsuno et al. reported that a lack of formal support from health care professionals as well as a lack informal support from family and friends was associated with postpartum depression during the pandemic [18]. This suggests that health care professionals may be required to provide emotional support for mothers as well as informational support such as how to breastfeed and how to respond to crying. Even in a situation where it is difficult to go out freely due to the pandemic, ongoing efforts are needed to prevent mothers from isolating themselves, including home visits, infant counseling services, and maintaining opportunities to interact with other mothers, whether face to face or by using SNS and/or video chat services. During the COVID-19 pandemic, home visit services were difficult to perform and consultations were sometimes conducted by telephone. Isaka et al. reported that 15% of women who gave birth between January and October 2020 refused home visits [16]. However, home visits are a valuable opportunity to meet both the mother and child at home and assess the risk of postpartum depression and child maltreatment [28]. Although phone calls, email, and social networking services (SNS) might play an increasingly important role, it is important to encourage mothers to allow home visits even during a pandemic.

In the present study, some participants felt stressed by having to refrain from direct contact with the children during home visits, which they routinely had before the pandemic, as well as refraining from providing breastfeeding care. They indicated a conflict between their desire to lighten the mother’s burden by holding the children and sympathizing with the mother’s feelings, even if for only a short time, and the pressure to prioritize infection control measures. Healthcare professionals have the requisite knowledge to support mothers and children during the postpartum period. For example, ensuring adequate sleep after childbirth is important for the mother’s physical and mental health. After childbirth, breastfeeding continues every 3 h, and sleep deprivation is common. Some studies have reported that sleep disturbances, sleep quality, and daytime arousal difficulties are associated with depression at 1 month postpartum [29]. However, during the pandemic, health care workers could not support mother to introduce mothers to facilities where they could rest because these facilities were closed. Therefore, health care workers have experienced anxiety and feelings of helplessness because of the pandemic, and it may be necessary to provide them with both physical and psychological support. Health care professionals involved in mental health support for mothers are likely to experience mental fatigue, which is known as “empathy fatigue,” as a result of their efforts to listen to narratives and support feelings in diverse cases [30]. In providing postpartum mental health support, it may be necessary to increase opportunities to interact with and listen to mothers at various places in the community, rather than leaving this role solely to health care professionals who make home visits. The present findings suggest that the creation of a system in which a wide variety of actors, including medical institutions and local childcare circles, provide multiple layers of care for mothers and children may prevent isolation in mothers and their children, reduce the psychological burden on the health care professionals who support them, and contribute to safe and secure childrearing in the community, not only during the pandemic but also in the post-pandemic world [31]. We believe that support for medical professionals will lead to better support for mothers. In the future, additional surveys of mothers should be conducted to determine what support is needed in the community.

### Limitations

To our knowledge, this is the first study to investigate the difficulties of health care professionals in supporting mothers and children during the COVID-19 pandemic. The perspectives identified in this study could be applied to post-pandemic support. Some limitations exist in this study. This study was based on group interviews with 13 health care professionals working in one municipality in the Kanto region of Japan, and thus the data are biased by the population size and regional characteristics of the municipality. Additional studies targeting health care professionals in other municipalities are needed in the future. In addition, because this study is based on interviews conducted from November to December 2021, it is possible that the difficulties experienced by health care professionals will change as the prevalence of COVID-19 changes. Further research should be conducted over the long term.

## Conclusion

This study identified difficulties faced by health care professionals in supporting mothers and infants in the community during the COVID-19 pandemic. Although these difficulties may have manifested during the pandemic, they may provide an important perspective for postpartum mental health support even after the pandemic ends. Therefore, it seems necessary for visiting professionals to receive mental health support through multidisciplinary collaboration. A novel aspect of this study suggests that better postpartum care would be realized by providing support that addresses the difficulties and challenges faced by the health care professionals who provide postpartum support for mothers. In addition, the findings of this study suggest the importance of collaboration among medical professionals and multidisciplinary professionals in improving postpartum care in the community.

### Definition of terms

Terms used in this study and their definitions are listed in Table 4.

**Table 4:** Definitions

Terms	Definitions
Health care professionals	Public health nurses, midwives, and nurses
COVID-19 pandemic	The ongoing global pandemic of coronavirus disease 2019 (COVID-19)
Partners	In our interviews, the mothers used a variety of names for their partners with whom they shared responsibility for the care of their children, including “father”, “dad”, and “husband”; in this study, all of these names were unified under the term “partners”.
Konnichiwa Akachan Jigyo	A welfare program implemented by each municipality in which home visits are made to families with infants within 4 months of birth [9].
Edinburgh Postpartum Depression Scale (EPDS)	A set of 10 screening questions that can indicate whether a mother has the symptoms common in women with depression and anxiety during pregnancy and in the year following the birth of a child [10, 11].
*Sato-gaeri bunben*	The traditional approach to childbirth in Japan. Mothers return to their parents’ home to prepare for childbirth and remain there to receive support while recovering physically and psychologically after giving birth [15].

## Data Availability

The datasets generated and/or analyzed during the current study are not publicly available in order to protect participants. Coding frames and the steps involved in the analysis are available from the corresponding author on reasonable request.

## List of abbreviations

COVID-19: coronavirus disease 2019
WHO: World Health Organization
EPDS: Edinburgh Postpartum Depression Scale
